# Screening outcomes at second FIT screening in individuals with a first time negative FIT‐result or low‐risk adenomas: Results from a nationwide FIT screening program

**DOI:** 10.1002/ijc.35419

**Published:** 2025-03-28

**Authors:** Pernille Thordal Larsen, Susanne Fogh Jørgensen, Morten Rasmussen, Berit Andersen, Sisse Helle Njor

**Affiliations:** ^1^ Department of Public Health Programmes, Randers Regional Hospital UNICCA—University Research Clinic for Cancer Screening Randers Denmark; ^2^ Department of Clinical Medicine Aarhus University Faculty of Health Sciences Aarhus Denmark; ^3^ Research Unit for Screening and Epidemiology, Department of Biochemistry and Immunology Lillebaelt Hospital—University Hospital of Southern Denmark Vejle Denmark; ^4^ Danish Colorectal Cancer Center South Lillebaelt Hospital—University Hospital of Southern Denmark Vejle Denmark; ^5^ Department of Regional Health Research University of Southern Denmark Odense Denmark; ^6^ Digestive Disease Center Bispebjerg Hospital Copenhagen Denmark; ^7^ Faculty of Health and Medical Sciences, Department of Clinical Medicine University of Copenhagen Copenhagen Denmark

**Keywords:** colorectal cancer, Faecal immunochemical test, low‐risk adenomas, screening

## Abstract

In Denmark, participants in faecal immunochemical test (FIT) screening with low‐risk adenomas are recommended a return to biennial FIT‐screening. However, they participate less than the FIT‐negative group (FIT_1_‐negative) at subsequent screening. Further, it is not clear how much this group benefits from the subsequent screening. We aimed at comparing the CRC incidence before and at the next screening (FIT_2_) in the low‐risk group to that of those having a FIT‐negative result at first time FIT‐screening. In this register‐based cohort study, we estimated the incidence of interval CRC (ICRC) and results of FIT_2_, including the FIT_2_‐positivity rate and rate of screen detected CRC (SDCRC). Relative risk (RR) comparing the low‐risk group to FIT_1_‐negatives was estimated. Adjustment for age and sex was performed with binary regression and presented with a 95% confidence interval (CI). Incidence of ICRC was 0.17% and 0.08% in the Low‐risk group and FIT_1_‐negative group, respectively, RR 2.18 (95%CI 1.51; 3.16). After adjustment, RR was 1.76 (95%CI: 1.22; 2.55). The FIT_2_‐positivity rate was 14.4% and 4.4% for the Low‐risk group and FIT_1_‐negative group, respectively. At FIT_2_‐screening, the detection of SDCRC was 0.36% and 0.16% in the low‐risk and FIT_1_‐negative group, respectively, RR 2.27 (95%CI: 1.46; 3.54), adjusted 1.83 (95% CI: 1.17; 2.85). Despite a recent colonoscopy, participants having low‐risk adenomas detected at first colonoscopy in FIT‐screening remain at a higher short‐term risk of ICRC and SDCRC compared to the FIT_1_‐negatives. Continuous participation in FIT‐screening is important for the Low‐risk group.

## Graphical abstract

What's new?

Fecal immunochemical test (FIT) screening is increasingly common as a population‐based screening tool for colorectal cancer (CRC). If the follow‐up colonoscopy after a positive FIT detects only low‐risk adenomas, those participants are recommended to continue biennial screenings, the same as people with negative FIT results. Here, the authors compared outcomes of the low‐risk group with those among the FIT‐negative group. They found that people with low‐risk adenomas had a higher short term risk of cancer than those who had an initial negative FIT. However, low‐risk participants were less likely than FIT‐negative participants to return a second FIT, which may have slightly affected the results of second FIT outcomes.

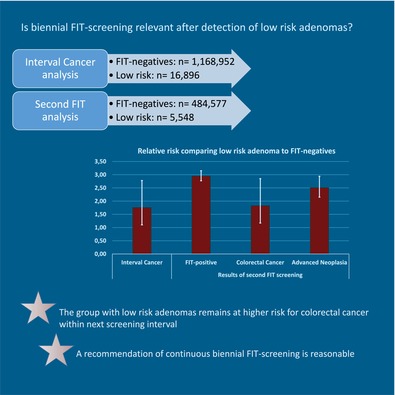

**Abbreviations**
ADR: Adenoma detection rate
AN: Advanced neoplasia
CI: Confidence interval
CRC: Colorectal cancer
DCCG.dk: Danish Colorectal Cancer Group database
ESGE: European Society of Gastrointestinal Endoscopy
FAP: Familial adenomatous polyposis
FIT: Faecal immunochemical test
IBD: Inflammatory bowel disease
ICRC: Interval colorectal cancer
IQR: Interquartile range
PCCRC: Post‐colonoscopy colorectal cancer
PPV: Positive predictive value
RR: Relative risk
RR‐PPV: Relative ratio of the PPV
SDCRC: Screen‐detected colorectal cancer
SNOMED‐dk: Systematized Nomenclature of Medicine—Danish version
SSL: Sessile serrated lesions
TNM: Tumour, node, metastasis
UICC: Union for International Cancer Control

## 1 INTRODUCTION

Bowel cancer screening programs have been implemented worldwide to reduce the morbidity and mortality of colorectal cancer (CRC), as the disease is the third most common cancer and accounts for the second most cancer‐related deaths.^1^, ^2^ To reduce the risk of recurrent or metachronous lesions, screening participants who get advanced neoplastic lesions (AN) detected at colonoscopy are recommended for colonoscopy surveillance according to several international guidelines.^3^, ^4^, ^5^ Meanwhile, participants who get non‐advanced neoplastic lesions detected are recommended to return to the ordinary screening program.^3^, ^5^

A retrospective cohort study has shown an average annual age‐adjusted CRC incidence in the low‐risk adenoma group comparable to the group with normal findings at colonoscopy.^6^ Other studies have shown that they have no benefits from colonoscopy surveillance.^7^, ^8^, ^9^ However, these studies are based on colonoscopies in non‐faecal immunochemical test (FIT) screening settings.

FIT screening is broadly being implemented as a population‐based bowel screening.^10^ With the fast implementation and increase in colonoscopy demand, evidence from non‐FIT‐based settings has been applied in FIT‐based screening settings. However, as the FIT‐positive population is a selected group, the evidence from primary colonoscopy screening and non‐screening settings may not be directly applicable to FIT screening.^11^ The impact of missed lesions might be higher in a FIT‐based setting.^11^, ^12^

In Denmark, the FIT‐positive individuals with low‐risk adenomas detected at colonoscopy are referred back to biennial FIT screening, similar to the FIT‐negative individuals.

We aimed to compare the screening outcomes of the low‐risk group to the FIT‐negative group after the first screening process. More specifically, we wanted to estimate the cumulated interval CRC (ICRC) incidence within biennial FIT screening and outcomes at the next FIT screening in terms of the FIT positivity rate, positive predictive values (PPV) and rate of screen‐detected CRC (SDCRC). If differences could be detected, healthcare resources might be used differently in order to secure an efficient screening program.

## 2 MATERIALS AND METHODS

### 2.1 Setting

The Danish FIT‐based bowel screening program was initiated in March 2014 and implemented during a 4‐year rollout. All Danish citizens aged 50–74 years were invited. During the rollout, screening intervals from 2 to 4 years were accepted in order to accommodate colonoscopy resources. Since 2018, the screening interval has been 2 years (±3 months).

Eligible citizens receive a FIT kit and invitation letter by mail informing and recommending them to participate by returning a faecal sample through mail. If the returned sample contains ≥20 μg haemoglobin/g faeces, the participant is, by law, offered a colonoscopy within 14 days.^13^

The FIT screening and all procedures related to screening and colonoscopy findings are free of charge as part of Denmark's tax‐payer‐funded universal health care system.^14^

In Denmark, screening participants with advanced neoplasia are enrolled in a surveillance program, while participants with a negative colonoscopy are quarantined from screening for 8 years.^15^

Until 2023, participants with low‐risk adenoma (1–2 adenomas, <10 mm and no villous components or high‐grade dysplasia) were referred back to the biennial screening program. Meanwhile, participants with more severe findings were referred to a surveillance colonoscopy program.^15^ Since 2023, participants having <5 adenomas, polyps <10 mm and no high‐grade dysplasia or sessile serrated lesions with dysplasia are recognised as being at low risk of CRC according to the 2020 guideline from the European Society of Gastroenterology (ESGE2020) and referred back to the biennial screening program.

### 2.2 Study design

The study was designed as a retrospective cohort study of prospectively collected register data. We included all screening participants aged 50–72 years residing in Denmark who received their first FIT screening between March 2014 and September 2019 with the result of either a Negative FIT (<20 μg haemoglobin/g faeces) or a positive FIT result with a subsequent colonoscopy showing only low‐risk findings. We did not include participants with a history of CRC, inflammatory bowel disease (IBD) or familial adenomatous polyposis (FAP). In a two‐step analysis, we followed the screening participants from the end of the first FIT (FIT_1_) screening process until invitation to the next FIT screening (FIT_2_) or 2 years after the FIT_1_, whichever came first. The outcome of interest of the first analysis was interval CRC (*ICRC analysis*).

In the *FIT*_*2*_ *analysis*, we included all screening participants with a screening interval of ≤2.5 years and estimated the FIT‐positivity rate, the positive predictive value for CRC and AN, and the detection rate of CRC and AN out of all FIT_2_‐screened.

Participants were excluded from the ICRC analysis if they were diagnosed with IBD or FAP, emigrated, or died before a diagnosis of CRC or the end of follow‐up. Participants were excluded from the FIT_2_ analysis if they had >2.5 years between the date of FIT_1_ and the invitation to FIT_2_; if they emigrated, died, were diagnosed with CRC or IBD/FAP before the FIT_2_ invitation, did not return the FIT_2_ within 6 months, or did not participate again.

### 2.3 Data sources

All residents in Denmark are given a unique 10‐digit identification number at birth or immigration. This allowed the combining of individual‐level register data from several registers.^14^ Screening participants were identified using the Danish Colorectal Cancer Screening Database from the Danish Clinical Quality Program.^16^ Here, we retrieved the date of the invitation and FIT result, as well as the FIT value. Information on colonoscopy procedures, diagnosis, colonoscopy results, and completeness of colonoscopy from both private and public hospitals was retrieved from the National Patient Register.^17^ Information on colonoscopy procedures performed at private clinics was retrieved from The National Health Service Register.^18^ Information on pathology specimens was retrieved from the Danish Pathology Register, which stores information on all pathology specimens retrieved in Denmark using the Danish version of the Systematized Nomenclature of Medicine (SNOMED‐dk).^19^ Cancer diagnoses were identified through the Danish Cancer Register and the Danish Colorectal Cancer Group Database (DCCG.dk).^20^ Information on birth, place of residence, death, or emigration was retrieved from Statistics Denmark.^14^

### 2.4 Definition of low‐risk group

The low‐risk group was defined as participants having a positive FIT and a colonoscopy within 3 months showing 1–2 adenomas with a size of <10 mm, no high‐grade dysplasia, and no villous histology. Sessile serrated lesions (SSL) with dysplasia were counted as adenomas. As some participants might need more than one colonoscopy due to incomplete index colonoscopy or incomplete resection of precursors, we grouped participants based on risk code (ZPY1E*) registered in the national patient register within 3 months from the first colonoscopy. If no risk codes were registered, we relied on data from the pathology register during the 3 months.

### 2.5 Definitions of CRC and AN

CRC cases were identified by Danish ICD‐10 codes of DC18*–DC20* registered in the Danish National Cancer Register or DCCG.dk, or any malignancy detected in or emerging from the colorectum, registered in the pathology register (SNOMED‐dk: M8*3 combined with location in the colorectum [T67*–T68*, T65900‐2, T65926], or M8*4/6 combined with ÆF4550 [emerging from the colorectum]). CRC was categorised as proximal if located from the cecum to the splenic flexure, distal if located in the descending colon to the rectosigmoid colon, or rectal. Cancer stages were categorised as UICC stages I–IV based on the TNM‐Classification (Shift from fifth to 8th edition in 2017).^21^

CRC cases were defined as Interval CRC (ICRC) if detected between the end of the FIT_1_ screening process and the invitation to FIT_2_ or 2 years after FIT_1_, whichever came first. This is in accordance with the World Endoscopy Organisations Consensus Statements on Post‐Colonoscopy and Post‐imaging Colorectal Cancer.^22^ The FIT_1_ screening process ended on the day of the FIT_1_ result for the FIT_1_‐negative group, while cancers detected within 6 months from the first FIT_1_‐derived colonoscopy were counted as FIT_1_‐screen detected cancers for the FIT_1_‐positive population. Defining cancer cases detected within 6 months from initial screen‐derived colonoscopy as screen‐detected CRC was in line with the WEO consensus statement on post‐colonoscopy CRC (PCCRC).^22^

CRC cases were defined as SDCRC if detected within 6 months of a positive FIT_2_ result. It was not a criterion that they had a colonoscopy, as this might not be possible for all cancer cases.

AN was defined as an SDCRC or polyps requiring surveillance detected within 3 months from the first FIT_2_‐screen‐derived colonoscopy. ESGE2020 defines polyps requiring surveillance as polyps ≥10 mm, adenomas with high‐grade dysplasia, sessile serrated lesions with dysplasia, or ≥5 adenomas.^3^ The piecemeal technique is used for the removal of large polyps, but they do not have a pathology‐registered size. Thus, all polyps removed with piecemeal polypectomy were interpreted as polyps ≥10 mm.

### 2.6 Statistical analysis

Baseline characteristics of sex and age of both the ICRC‐analysis and the FIT_2_‐analysis were presented. Age was categorised into five age groups: 49–54, 55–59, 60–64, 65–69, 70 to <73.

The 2‐year cumulated ICRC incidence in both the FIT_1_‐negative and low‐risk group was estimated, and the Relative Risk (RR) of ICRC was presented by comparing the Low‐risk group to the FIT_1_‐negative group.

The FIT_2_‐positivity rate was calculated as the proportion of positive FIT_2_ results out of all valid FIT_2_ results. The RR of a positive FIT_2_ was compared between the groups.

The positive predictive value (PPV) of FIT_2_ was calculated for both CRC and AN as the proportion with SDCRC/AN out of all FIT‐positives having a colonoscopy follow‐up or SDCRC. We estimated the Relative Ratio of the positive predictive values (RR‐PPV) of FIT_2_ in the low‐risk group compared to the FIT_1_‐negative group.

The SDCRC and AN detection was calculated as the proportion of FIT_1_‐screened individuals having SDCRC and AN at the second screening.

Using binary regression, we adjusted relative estimates for sex and age groups, as these are well‐known factors that influence CRC screening outcomes.^23^ All estimates were presented with a 95% Confidence interval (CI). Analyses were conducted using STATA statistical software version 17 (StataCorp., College Station, TX).

Sensitivity analyses: In sensitivity analyses, we: (i) only included low‐risk participants with agreement between pathology data and the endoscopist registered risk and (ii) Only included low‐risk participants registered with a code for “complete colonoscopy”, meaning there was cecal intubation and a clear view of the mucosa (i.e., sufficient bowel prep). The Negative Predictive Value of a registration of “Completeness of Colonoscopy” was 20% in the first year of the screening program according to a validation study of the screening database.^16^ Thus, excluding them could result in selection bias. However, the sensitivity analysis should clarify, if incomplete colonoscopies were not a dominating factor for the results; (iii) Excluded individuals with SSL with dysplasia, as these could have been risk stratified as low risk if <10 mm according to the old Danish guideline. However, this is not in accordance with neither the 2013 and 2020 guideline from ESGE.^3^, ^24^ In order to ensure the applicability of the results to newer guidelines, we needed to ensure the results were not driven by the SSL with dysplasia.

## 3 RESULTS

### 3.1 ICRC analysis

**FIGURE 1:**
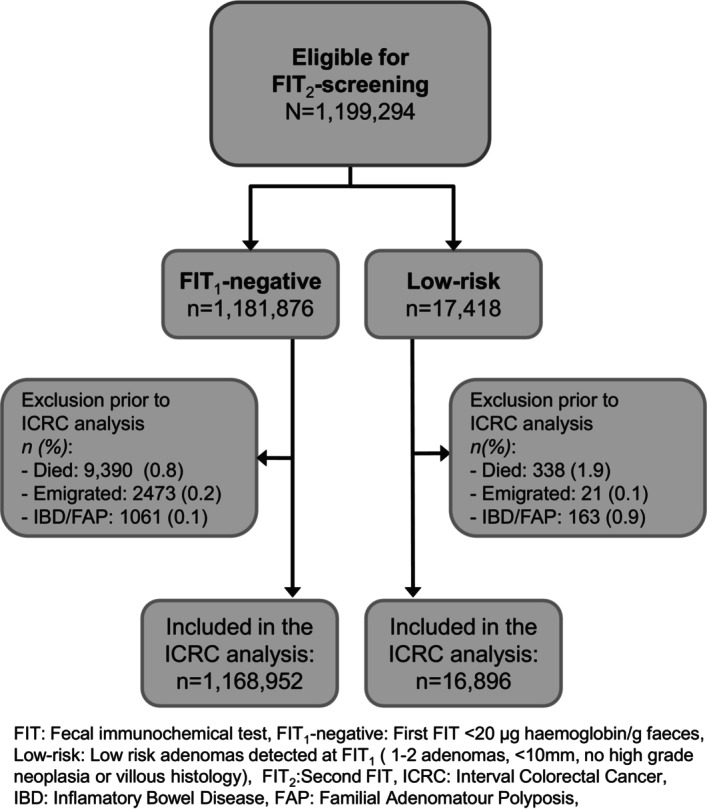
Flowchart of inclusion and exclusion in interval colorectal cancer analysis.

Initially, we included 1,181,876 screening participants with a negative FIT_1_ and 17,418 with a low‐risk result. A larger proportion of participants were excluded due to diagnosis with IBD/FAP or death in the low‐risk group than in the FIT_1_‐negative group. After exclusion, 1,168,952 FIT_1_‐negative participants and 16,896 with a low‐risk result were included in the ICRC analysis (Figure 1).

The low‐risk group had a majority of men, while the FIT_1_‐negative group had a majority of women. The low‐risk group was generally older than the FIT_1_‐negative group (Table 1). In the low‐risk group, 93.6% had a registration of complete colonoscopy, and 0.4% had SSL with dysplasia detected.

**TABLE 1:** Baseline information in interval CRC analysis and FIT_2_‐analysis.

	Interval CRC analysis	FIT_2_‐analysis		
	FIT_1_‐negative	Low‐risk	FIT_1_‐negative	Low‐risk
	*n* = 1,168,952	*n* = 16,896	*n* = 484,577	*n* = 5548
Sex, *n* (%)				
Male	537,086 (46.0)	9865 (58.4)	219,850 (45.4)	3088 (55.7)
Female	631,866 (54.1)	7031 (41.6)	264,727 (54.6)	2460 (44.3)
Age group, *n* (%)				
49–54	379,283 (32.5)	3466 (20.5)	144,033 (29.7)	1011 (18.2)
55–59	229,145 (19.6)	2782 (16.5)	98,371 (20.3)	911 (16.4)
60–64	219,559 (18.8)	3653 (21.6)	97,217 (20.1)	1308 (23.6)
65–69	221,004 (18.9)	4375 (25.9)	95,568 (19.7)	1488 (26.8)
70–73	119,961 (10.3)	2620 (15.5)	49,388 (10.2)	830 (15.0)
Age, *Median* (IQR)	59.4 (53.1; 66.2)	63,1 (56.4; 68.3)	60.0 (53.7; 66.3)	63.4 (57.2; 68.1)
Registration of complete colo^a^		15,820 (93.6)		5141 (92.7)
SSL with dysplasia^b^		72 (0.4)		35 (0.6)

*Note*: *FIT2*: *Second* Faecal immunochemical test (FIT)—invitation <2.5 years from day of first FIT. *FIT*_*1*_‐*negative*: Screening participants with a first time FIT <20 μg haemoglobin/g faeces. *Low*‐*risk*: FIT_1_‐positives and colonoscopy showing 1–2 adenomas, <10 mm, no high grade dysplasia and no villous‐adenomas.

Abbreviation: IQR, interquartile range.

^a^ Registration of complete colonoscopy at baseline defined as a colonoscopy reaching ceacum and with clear view of the mucosa.

^b^ Sessile Serrated Lesions with dysplasia <10 mm could be stratified into low risk according to old Danish guideline.

**FIGURE 2:**
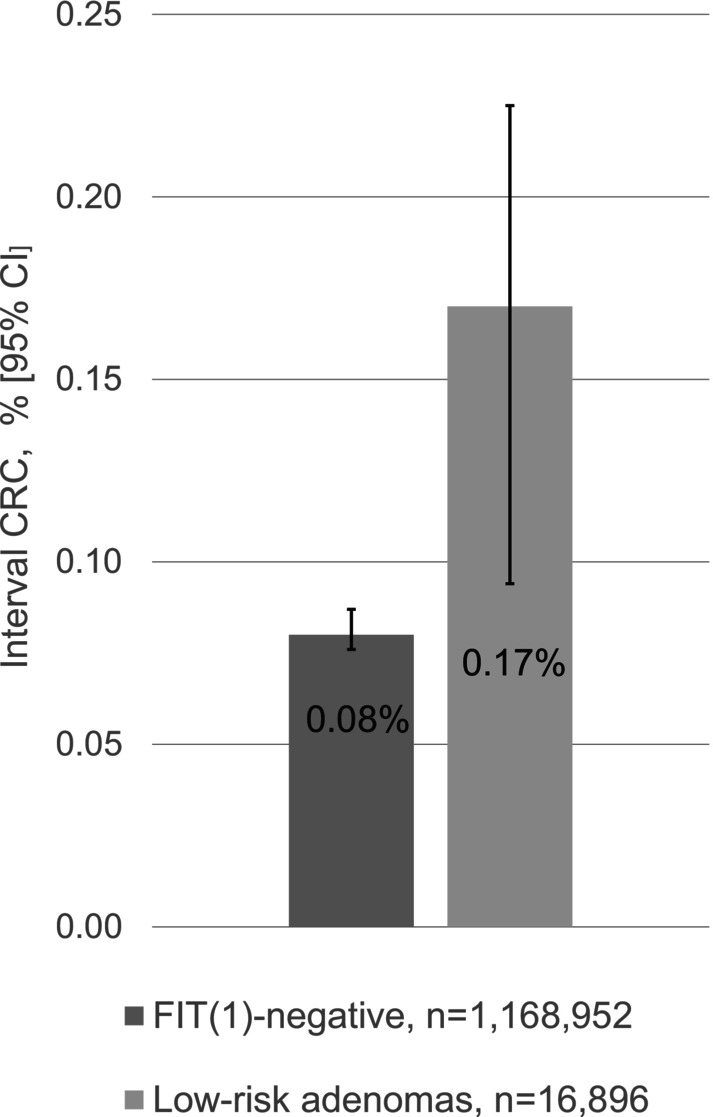
Two‐year cumulated interval colorectal cancer incidence by result of first screening.

Within 2 years, 920 FIT_1_‐negative individuals were diagnosed with ICRC, while 29 individuals were diagnosed in the low‐risk group (Figure 2). This resulted in a crude RR of 2.18 (95% CI: 1.51; 3.16). After adjustment for sex and age, the RR was 1.76 (95%CI: 1.22; 2.55).

### 3.2 FIT_2_ analysis

**FIGURE 3:**
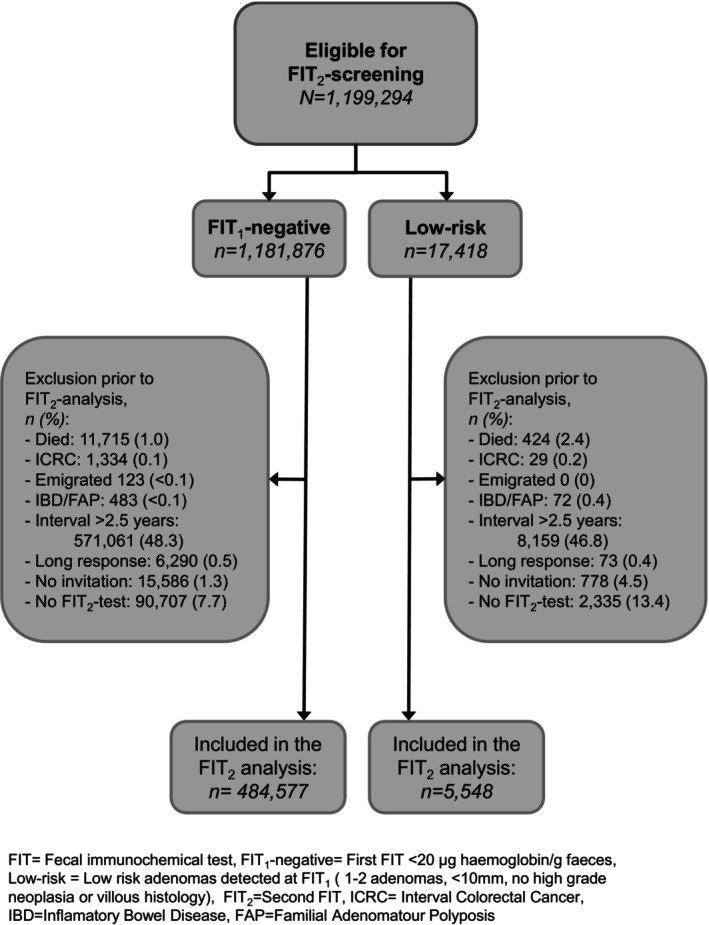
Flowchart of inclusion and exclusion in FIT_2_ analysis.

The Low‐risk group had a larger proportion of non‐invited (4.5% and 1.3%), non‐participants in FIT_2_ (13.4% vs. 7.7%) and individuals with ICRC, IBD, and FAP (Figure 3). Finally, 484,577 of the FIT_1_‐negative and 5548 of the low‐risk group were included in the FIT_2_ analysis (Figure 3).

The FIT_2_ analysis also had a majority of males in the low‐risk group, a majority of women in the FIT_1_‐negative group, and the low‐risk group was older than the FIT_1_‐negative group (Table 1). In the low‐risk group, 92.7% had a registration of complete colonoscopy and 0.6% had SSL with dysplasia at baseline colonoscopy.

The FIT_2_ positivity rate was 4.4% for the FIT_1_‐negatives (95%CI: 4.3; 4.5), while 14.4% for the low‐risk group (95% CI 13.5; 15.4), leaving an RR of 3.28 (95% CI; 3.08; 3.51; Table 2). The positive predictive value for CRC was 3.9% in the FIT_1_‐negative group, while 2.7% in the low‐risk group, leaving an RR‐PPV of 0.70 (95% CI: 0.45; 1.09). After adjustment, the RR‐PPV was 0.64 (95%CI: 0.41; 0.98).

**TABLE 2:** Results of second FIT screening comparing low‐risk to FIT_1_‐negatives.

	FIT_1_‐negative		Low‐risk			
	*n*	%	*n*	%	RR, crude	RR, Adj^a^
		(95% CI)		(95% CI)	(95%CI)	(95% CI)
FIT_2_ participants	484,577		5548			
SDCRC	768	0.16	20	0.36	2.27	1.83
		(0.15; 0.17)		(0.22; 0.56)	(1.46; 3.54)	(1.17; 2.85)
AN	4628	0.96	160	2.88	3.02	2.51
		(0.93; 0.98)		(2.46; 3.36)	(2.59; 3.53)	(2.15; 2.94)
FIT_2_ positives	21,298	4.4	801	14.4	3.28	2.95
		(4.3; 4.5)		(13.5; 15.4)	(3.08; 3.51)	(2.77; 3.15)
No colonoscopy	1623		70			
PPV‐CRC	768	3.90	20	2.74	0.70	0.64
		(3.63; 4.18)		(1.68; 4.19)	(0.45; 1.09)	(0.41; 0.98)
PPV‐AN	4628	23.52	160	21.89	0.93	0.86
		(22.93; 24.12)		(18.94; 25.06)	(0.81; 1.07)	(0.75; 0.99)

*Note*: *FIT*_*1*_‐*negative*: Screening participants with a first time Faecal immunochemical test (FIT) <20 μg haemoglobin/g faeces. *Low*‐*risk*: FIT_1_‐positives and colonoscopy showing 1–2 adenomas, <10 mm, no high grade dysplasia and no villous‐adenomas. *SDCRC*: Screen detected colorectal cancer = CRC diagnosed within 180 days from positive FIT. *FIT*_*2*_: Second FIT‐screening. SD‐*AN*: Screen detected advanced neoplasia = polyps requiring surveillance according to ESGE2020 guideline detected within 3 months from a positive FIT (polyps ≥10 mm, ≥5 adenomas, high grade dysplasia or sessile serrated lesions with dysplasia) or CRC.

^a^ Adj: adjusted for sex and age group (49–54, 55–59, 60–64, 65–69, and 70 to <73).

When estimating the proportion of SDCRC out of all FIT_2_ participants, 0.36% (95%CI: 0.22; 0.56) of the Low‐risk group had SDCRC, while 0.16 % (95% CI: 0.15; 0.17) of the FIT_1_‐negative group had SDCRC. This resulted in a crude RR of 2.27 (95% CI: 1.46; 3.54) of SDCRC in the Low‐risk group compared to the FIT_1_‐negative group, and after adjustment, the risk was still significantly increased, RR 1.83% increased risk (95%CI: 1.17; 2.85; Table 2).

Of all FIT_2_‐screened, 0.96% (95%CI 0.93; 0.98) of the FIT_1_‐negatives had AN detected, while 2.88% (95%CI 2.46; 3.36) of the low‐risk group, resulting in a crude three‐fold increased risk of AN detection for the low‐risk compared to FIT_1_‐negatives. After adjustment, the RR was 2.51 (95%CI: 2.15; 2.94). (Table 2) The PPV for AN was 23.5% in the FIT_1_‐negative group and 21.9% in the Low‐risk group, leaving an RR of 0.93 (95% CI: 0.81; 1.07). After adjustment, this estimate became statistically significant, RR 0.86 (95% CI: 0.75; 0.99).

For the ICRC analysis and the FIT_2_ analysis, there was no significant difference in estimates when only including Low‐risk participants with complete agreement between pathology data and endoscopist registration (data not shown). When excluding individuals without a registration of complete colonoscopy, the estimates did in general not change in neither the ICRC analysis nor the FIT_2_ analysis (data not shown). However, the adjusted RR of SDCRC were reduced to 1.50 (95% CI: 0.96; 2.58).

The number of CRC cases among individuals with SSL with dysplasia was too few to report according to Danish legislation, and excluding them did not change the RR estimates significantly in any of the analyses (Data not shown).

### 3.3 CRC case details

The ICRC cases were detected as stage III–IV in 56.8% and 62.1% of cases in the FIT_1_‐negative and Low‐risk groups, respectively (Table 3). Meanwhile, both groups had a majority of SDCRC cases detected at stage I–II, that is, 65.2% in the FIT_1_‐negative group and 55% in the low‐risk group. Proximal cancers were more frequent in the FIT_1_‐negative group, with a proportion of 47.4% and 40% of ICRC and SDCRC (Table 3).

**TABLE 3:** Details on colorectal cancer (CRC) cases.

	FIT1‐negative				Low‐risk			
	ICRC *n* = 920		SDCRC *n* = 768		ICRC *N* = 29		SDCRC *n* = 20	
UICC stage	*n*	%	*n*	%	*n*	%	*n*	%
I	205	23.9	345	45.1	5	17.2	8	40.0
II	181	20.3	154	20.1	6	20.7	3	15.0
III	233	26.1	216	28.2	8	27.6	5	25.0
IV	274	30.7	50	6.5	10	34.5	4	20.0
Unknown	27	N/A	3	N/A	0	N/A	0	N/A
Location								
Proximal colon	427	47.4	307	40.2	10	34.5	6	30
Distal colon	178	19.8	211	27.6	^a^	<27.5	7	35
Rectal	296	32.9	246	32.2	11	38	7	35
Unknown	19	N/A	4	N/A	^a^	N/A	0	N/A

*Note*: FIT_1_‐negative: Screening participants with a first time Faecal immunochemical test (FIT) <20 μg haemoglobin/g faeces. Low‐risk: FIT_1_‐positives and colonoscopy showing 1–2 adenomas, <10 mm, no high grade dysplasia and no villous‐adenomas, ICRC: Interval CRC, SDCRC: Screen detected CRC. UICC stages based on 5th and 8th edition (Shift in classification in Denmark in 2017). Location: Proximal (Cecum to splenic flexure), Distal (Descending‐ and sigmoid colon).

Abbreviation: N/A, non‐applicable.

^a^ Exact numbers cannot be presented due to numbers in unknown<3.

The sample size was too small to test for statistically significant trends in site and stage distribution for either SDCRC or ICRC between groups.

## 4 DISCUSSION

### 4.1 Main findings

In this nationwide cohort study, we compared the ICRC risk and outcome at the next FIT screening between those who had a negative FIT result at the prevalence FIT screening and those who had low‐risk adenomas detected. We found a more than two‐fold increased risk of ICRC and a 3‐fold increased risk of having a positive FIT at the second screening for the Low‐risk group compared to the FIT_1_‐negative group. After adjusting for age and sex, a prior screening result of low‐risk adenomas was still associated with a 76% increased risk of ICRC and a 3‐fold risk of having a positive FIT_2_. Compared to the FIT_1_‐negative group, the low‐risk group had a crude 30% non‐significantly lower PPV for CRC and a similar PPV for AN. This only changed marginally but became statistically significant when adjusting for sex and age.

### 4.2 Comparisons with other studies

To our knowledge, this is the first study to directly compare the next screening outcome in the Low‐risk adenoma group to the FIT_1_‐negative group in a biennial FIT‐screening program with cut‐offs at ≥20 μg haemoglobin/g faeces.

Our results on ICRC rates in the FIT_1_‐negative group were in line with the annual report from the Danish Cancer screening database from 2022,^25^ and as expected, lower than the 10.4 per 10.000 screened reported in the Dutch FIT‐screening program, which has a cut‐off at ≥47 μg haemoglobin/g faeces.^26^ For the Low‐risk group, a prior estimate on 2‐year cumulated ICRC of 0.11% was reported by Nielsen et al. based on Danish data.^27^ However, Nielsen et al. relied solely on risk codes from the Danish Colorectal Cancer Screening Database for risk group allocation, DCCG.dk for CRC cases, and their low‐risk sample size was smaller than in the present study (*n* = 6123 vs. *n* = 16,896). We cross‐checked risk group allocation with the pathology register due to the documented low validity of risk codes in the first years of the screening program^16^ and supplemented DCCG.dk data with the cancer register and pathology data in order to ensure all cases were included, as misclassification can have a large impact due to the low absolute numbers.

A Norwegian study from 2023 found a FIT_2_‐CRC detection rate of 0.25% among participants, who had a FIT_1_‐negative result (cut‐off at ≥15 μg haemoglobin/g faeces). Their detection rate was higher, than in the present study (0.16%), which is to be expected with a lower cut‐off value and thereby a larger proportion having follow‐up with colonoscopy.^28^

The PPV for CRC in the low‐risk group was 2.7%, which is similar to the PPV of 2.8% for CRC found in the Scottish Bowel Screening program with a FIT cut‐off at ≥80 μg haemoglobin/g faeces. A similar PPV for CRC despite the four times higher cut‐off can be due to either small numbers in the Scottish study (91 FIT‐positives) or the fact that Denmark has been shown to have a higher PCCRC‐rate than countries such as Sweden and England^29^ resulting in a higher PPV‐CRC at second screening for the low‐risk group.

Given the natural history of CRC, both ICRC and SDCRC in the Low‐risk group most likely represent missed lesions (CRCs or premalignant lesions).^22^ Despite our findings of a higher overall incidence in the Low‐risk group compared to the FIT_1_‐negative group, it is likely that they, during a long‐term follow‐up, would end up having an incidence closer to the FIT_1_‐negative population. A recent study was published on CRC incidence among screening participants with low‐ and high‐risk adenomas detected in an Italian FIT‐based screening program.^30^ They found no differences in the 15‐year cumulative incidence between screened individuals with Low‐risk adenomas and those with only FIT‐negative results.^31^

### 4.3 Strengths and limitations

The main strengths of our study are the nationwide design, the use of individual‐level data, and the combination of pathology and hospital records, which ensured several sensitivity analyses to ensure the robustness of the results. While a substantial part of first‐time screened individuals did not participate in FIT_2_, our result represents what is gained before and at the second screening for the FIT_1_‐negative and Low‐risk group. Our crude estimates represent the outcomes of the risk stratifications that do not take age, FIT numerical value, or sex into account. Meanwhile, our sex‐ and age‐adjusted estimates illustrate that the detected differences cannot be explained by age and sex alone. However, our study design and exclusion do not allow for conclusion on causal relations.

Certain limitations need attention: (1) We had to exclude approximately 50% of the screened population in the FIT_2_ analysis as they had a screening interval of >2.5 years. As allocation to screening interval was solely based on colonoscopy capacity and not FIT_1_ result, sex, or age, we found it best to only include those receiving a screening interval comparable to what is currently recommended. With a larger sample size, we would have been able to stratify results based on age and gender, which might have revealed subgroup differences in RRs.

The proportion of non‐participants at FIT_2_ was larger in the low‐risk group than in the FIT_1_‐negative group. While healthy‐user bias is a well‐known phenomenon within observational studies of preventive interventions, we do not know how this relation is at second screening. Thus, is unclear if those not participating is of higher or lower risk of CRC. This conclusion would need a long‐term follow‐up.

We did not have information on individual‐level endoscopist ADRs. The Danish screening program had an overall acceptable adenoma detection rate (ADR) of 49%–52% in the study period. In comparison, a FIT‐based screening program from north‐eastern Italy with a ≥20 μg haemoglobin/g faeces cut‐off had a mean ADR of 48%.^32^ However, a study from the Central Denmark Region found a significant variation in individual‐level ADR.^33^ With relatively few cases, few endoscopists with low ADRs could potentially increase the PCCRC incidence. We could not conclude on this, as there is no nationwide accessible registration of individual‐level ADR. However, the results represent the real‐life screening program in Denmark and the procedures the screening participants receive.

Due to the shift in guidelines, our low‐risk group will only partially represent the future low‐risk population, as it will now include some of the prior intermediate‐risk population (3–4 adenomas or villous adenomas <10 mm). In a recently published study, we have described this population.^34^ We extracted <73‐year‐olds from this cohort (*n* = 2732) and registered eight ICRCs within 2 years (0.29% [95% CI: 0.13; 0.58]). This is not statistically significantly different from the low‐risk group described in the present study, but the point estimate is higher. Thus, the RR of ICRC presented might be a slight underestimation of the true RR in the future. We cannot conclude how the new guidelines will affect the FIT_2_‐positivity rate and AN and CRC detection rate, as the prior intermediate group did not receive a FIT_2_. Meanwhile, individuals with small SSLs with dysplasia will now be considered in need of colonoscopy surveillance. However, the proportion with SSL with dysplasia in the low‐risk group was small and excluding them did not change any results substantially.

### 4.4 Implications

The higher incidence of ICRC in the low‐risk group, despite their recent colonoscopy, emphasizes the importance of high‐quality colonoscopy in the FIT‐based screening programs and a need to ensure acceptable ADRs for all screening endoscopists. The FIT‐positive population has a higher pre‐test probability of CRC, which affects the negative predictive value for CRC of the colonoscopy. With the FIT having lower sensitivity for less severe findings,^35^ it is likely that some of the detected lesions were not the cause of the positive FIT. This aligns with our previously published result showing a higher 3‐year cumulative CRC risk among the intermediate‐risk group with less severe findings at initial colonoscopy.^34^ Meanwhile, the lower ICRC risk in the FIT_1_‐negative group who have not received a recent colonoscopy speaks well for the negative predictive value of the FIT‐based screening program.

In the FIT_1_‐negative group, 631 (95%CI 598; 679) individuals needed to undergo FIT_2_ screening in order to find one case of CRC, while 105 (95% CI 102; 108) were required to detect one case of AN. Meanwhile, 277 (95%CI 193; 492) and 35 (95%CI 30; 41) in the Low‐risk group needed to undergo FIT_2_ screening to detect CRC and AN, respectively. Thus, the benefit from the FIT_2_‐screening was higher in the Low‐risk group. Contrary, nearly 37 (95% 26; 59) needed to have a colonoscopy to find one case of CRC in the low risk group, while only 26 (95% CI 24; 28) in the FIT_1_‐negative group. However, the yield in AN detection was similar, with 4.6 (95%CI 4.0; 5.3) needed to be scoped to detect one case of AN in the Low‐risk group and 4.3 (95% CI 4.2; 4.4) in the FIT_1_‐negative group. In our recent study looking into the yield during 3‐year colonoscopy surveillance for individuals with advanced neoplasia, approximately 100 and eight surveillance colonoscopies were needed in order to find one case of CRC and AN, respectively.^34^ In comparison, the harm in terms of unnecessary colonoscopies is thus, significantly lower in the low‐risk group receiving biennial FIT‐screening.

The participation rate at subsequent FIT screening among low‐risk groups is lower than in the FIT_1_‐negative population.^36^ Thus, it is of great importance to inform the participants with low‐risk adenomas of the gain in continuous screening participation. However, given the relatively low PVV for CRC in the low‐risk group, it needs to be monitored if they keep having a markedly higher FIT‐positivity rate, as this may result in a significant number of unnecessary colonoscopies during several screening rounds.

In conclusion, the screening participants with a Low‐risk result at first FIT screening remain at a higher risk of CRC within the first screening interval, and the importance of continuing to participate in FIT screening should be emphasized. Further studies are needed to conclude on multiple screening rounds.

## AUTHOR CONTRIBUTIONS

**Pernille Thordal Larsen:** Conceptualization; investigation; funding acquisition; writing – original draft; methodology; validation; visualization; writing – review and editing; formal analysis; project administration; data curation. **Susanne Fogh Jørgensen:** Writing – review and editing; data curation; supervision; validation. **Morten Rasmussen:** Supervision; writing – review and editing. **Berit Andersen:** Resources; supervision; writing – review and editing; funding acquisition. **Sisse Helle Njor:** Conceptualization; investigation; writing – review and editing; methodology; formal analysis; supervision; resources; funding acquisition; validation.

## CONFLICT OF INTEREST STATEMENT

Berit Andersen is head of the colorectal cancer screening program in Central Denmark Region and a member of the National Steering committee for Colorectal cancer screening and the Danish Colorectal Cancer Screening Database, but has no other conflicts of interest. Morten Rasmussen is head of the colorectal cancer screening program in the capital Region, head of the Colorectal Cancer Screening Database, and a member of the National Steering Committee for Colorectal cancer screening and has received speaking fees from Norgine for giving speeches at sponsored meetings. The money obtained through this has not been used to finance this manuscript. Sisse Helle Njor, Susanne Fogh Jørgensen, and Pernille Thordal Larsen declare no conflict of interest.

## ETHICS STATEMENT

According to the EU's General Data Protection Regulation (article 30), the project was listed at the record of processing activities for research projects in the Central Denmark Region (J. No.: 1‐16‐02‐429‐19). According to the Consolidation Act on Research Ethics Review of Health Research Projects, Consolidation Act number 1083 of 15 September 2017 section 14, notification of questionnaire surveys or medical database research projects to the research ethics committee system is only required if the project involves human biological material. Therefore, this study may be conducted without approval from the committees.

## DISCLOSURE

The originally submitted manuscript was included in the PhD thesis of P. T. Larsen.

## Data Availability

The data that support the findings of this study are available from Statistics Denmark and from The Danish Clinical Quality Program (The Danish colorectal Cancer Screening Database and The Danish Colorectal Cancer Group Database). Restrictions apply to the availability of these data, which were used under license for this study. Data may be available upon reasonable request to Statistics Denmark and The Danish Clinical Quality Program. Further information is available from the corresponding authors upon request.

## References

[1] Sung H , Ferlay J , Siegel RL , et al. Global cancer statistics 2020: GLOBOCAN estimates of incidence and mortality worldwide for 36 cancers in 185 countries. CA Cancer J Clin. 2021;71:209‐249. 33538338 10.3322/caac.21660

[2] Bénard F , Barkun AN , Martel M , von Renteln D . Systematic review of colorectal cancer screening guidelines for average‐risk adults: summarizing the current global recommendations. World J Gastroenterol. 2018;24:124‐138. 29358889 10.3748/wjg.v24.i1.124 PMC5757117

[3] Hassan C , Antonelli G , Dumonceau JM , et al. Post‐polypectomy colonoscopy surveillance: European Society of Gastrointestinal Endoscopy (ESGE) guideline: update 2020. Endoscopy. 2020;52:687‐700. 32572858 10.1055/a-1185-3109

[4] Gupta S , Lieberman D , Anderson JC , et al. Recommendations for follow‐up after colonoscopy and polypectomy: a consensus update by the US multi‐society task force on colorectal cancer. Gastrointest Endosc. 2020;91: 463–85.e5. 10.1016/j.gie.2020.01.014 PMC7389642 32044106

[5] Rutter MD , East J , Rees CJ , et al. British Society of Gastroenterology/Association of Coloproctology of Great Britain and Ireland/Public Health England post‐polypectomy and post‐colorectal cancer resection surveillance guidelines. Gut. 2020;69:201‐223. 31776230 10.1136/gutjnl-2019-319858 PMC6984062

[6] Lee JK , Jensen CD , Levin TR , et al. Long‐term risk of colorectal cancer and related death after adenoma removal in a large, community‐based population. Gastroenterology. 2020;158: 884–94.e5. 10.1053/j.gastro.2019.09.039 PMC7083250 31589872

[7] Wieszczy P , Kaminski MF , Franczyk R , et al. Colorectal cancer incidence and mortality after removal of adenomas during screening colonoscopies. Gastroenterology. 2020;158: 875–83.e5. 10.1053/j.gastro.2019.09.011 31563625

[8] Atkin W , Wooldrage K , Brenner A , et al. Adenoma surveillance and colorectal cancer incidence: a retrospective, multicentre, cohort study. Lancet Oncol. 2017;18:823‐834. 28457708 10.1016/S1470-2045(17)30187-0 PMC5461371

[9] Cottet V , Jooste V , Fournel I , Bouvier AM , Faivre J , Bonithon‐Kopp C . Long‐term risk of colorectal cancer after adenoma removal: a population‐based cohort study. Gut. 2012;61:1180‐1186. 22110052 10.1136/gutjnl-2011-300295

[10] Shaukat A , Levin TR . Current and future colorectal cancer screening strategies. Nat Rev Gastroenterol Hepatol. 2022;19:521‐531. 35505243 10.1038/s41575-022-00612-y PMC9063618

[11] van de Schootbrugge‐Vandermeer HJ , Kooyker AI , Wisse PHA , et al. Interval post‐colonoscopy colorectal cancer following a negative colonoscopy in a fecal immunochemical test‐based screening program. Endoscopy. 2023;55:1061‐1069. 37793423 10.1055/a-2136-6564 PMC10684335

[12] Peng SM , Hsu WF , Wang YW , et al. Faecal immunochemical test after negative colonoscopy may reduce the risk of incident colorectal cancer in a population‐based screening programme. Gut. 2021;70:1318‐1324. 32989019 10.1136/gutjnl-2020-320761 PMC8223654

[13] Njor SH , Friis‐Hansen L , Andersen B , et al. Three years of colorectal cancer screening in Denmark. Cancer Epidemiol. 2018;57:39‐44. 30292899 10.1016/j.canep.2018.09.003

[14] Schmidt M , Schmidt SAJ , Adelborg K , et al. The Danish health care system and epidemiological research: from health care contacts to database records. Clin Epidemiol. 2019;11:563‐591. 31372058 10.2147/CLEP.S179083 PMC6634267

[15] Rasmussen MI , Linnemann D , Larsen OB , Bang S . Screenings: og adenomkontrol program for tyk—og endetarmskræft. Vol 2019. Danish Regions; 2014.

[16] Thomsen MK , Njor SH , Rasmussen M , et al. Validity of data in the Danish colorectal cancer screening database. Clin Epidemiol. 2017;9:105‐111. 28255255 10.2147/CLEP.S124454 PMC5322846

[17] Schmidt M , Schmidt SA , Sandegaard JL , Ehrenstein V , Pedersen L , Sorensen HT . The Danish National Patient Registry: a review of content, data quality, and research potential. Clin Epidemiol. 2015;7:449‐490. 26604824 10.2147/CLEP.S91125 PMC4655913

[18] Andersen JS , Olivarius Nde F , Krasnik A . The Danish National Health Service Register. Scand J Public Health. 2011;39:34‐37. 21775348 10.1177/1403494810394718

[19] Bjerregaard B , Larsen OB . The Danish pathology register. Scand J Public Health. 2011;39:72‐74. 21775357 10.1177/1403494810393563

[20] Ingeholm P , Gögenur I , Iversen LH . Danish colorectal cancer group database. Clin Epidemiol. 2016;8:465‐468. 27822086 10.2147/CLEP.S99481 PMC5094575

[21] Luca B , Federica M , Jasna M , et al. Eighth edition of the UICC classification of malignant Tumours: an overview of the changes in the pathological TNM classification criteria—what has changed and why? Virchows Arch. 2017;472:519‐531. 29209757 10.1007/s00428-017-2276-y

[22] Rutter MD , Beintaris I , Valori R , et al. World endoscopy organization consensus statements on post‐colonoscopy and post‐imaging colorectal cancer. Gastroenterology. 2018;155: 909–25.e3. 10.1053/j.gastro.2018.05.038 29958856

[23] Jodal HC , Klotz D , Herfindal M , et al. Long‐term colorectal cancer incidence and mortality after adenoma removal in women and men. Aliment Pharmacol Ther. 2022;55:412‐421. 34716941 10.1111/apt.16686

[24] Hassan C , Quintero E , Dumonceau J‐M , et al. Post‐polypectomy colonoscopy surveillance: European Society of Gastrointestinal Endoscopy (ESGE) guideline. Endoscopy. 2013;45:842‐864. 24030244 10.1055/s-0033-1344548

[25] Regionernes Kliniske Kvalitetsudviklingsprogram . Danish colorectal cancer screening database. Annual report 2022, January 1 to December 31, 2022. [Dansk Tarmkræftscreeningsdatabase, Årsrapport for 2022,‐1. januar til 31. december 2022]. 2024.

[26] Breekveldt ECH , Toes‐Zoutendijk E , van de Schootbrugge‐Vandermeer HJ , et al. Factors associated with interval colorectal cancer after negative FIT: results of two screening rounds in the Dutch FIT‐based CRC screening program. Int J Cancer. 2023;152:1536‐1546. 36444504 10.1002/ijc.34373 PMC10107864

[27] Nielsen JC , Ploug M , Baatrup G , Kroijer R . Risk of post colonoscopy colorectal cancer following screening colonoscopy with low‐risk or no adenomas: a population‐based study. Colorectal Dis. 2021;23(11):2932‐2936. doi:10.1111/codi.15886 34427981

[28] Ribe SG , Botteri E , Løberg M , et al. Impact of time between faecal immunochemical tests in colorectal cancer screening on screening results: a natural experiment. Int J Cancer. 2023;152:1414‐1424. 36346118 10.1002/ijc.34351 PMC10098820

[29] Pedersen L , Valori R , Bernstein I , Lindorff‐Larsen K , Green C , Torp‐Pedersen C . Risk of post‐colonoscopy colorectal cancer in Denmark: time trends and comparison with Sweden and the English National Health Service. Endoscopy. 2019;51:733‐741. 31174223 10.1055/a-0919-4803

[30] Zorzi M , Battagello J , Amidei CB , et al. Low colorectal cancer risk after resection of high‐risk pedunculated polyps. Clin Gastroenterol Hepatol. 2024;22: 1518–1527.e7. 10.1016/j.cgh.2024.01.027 38325601

[31] Zorzi M , Antonelli G , Barbiellini Amidei C , et al. Adenoma detection rate and colorectal cancer risk in fecal immunochemical test screening programs: an observational cohort study. Ann Intern Med. 2023;176:303‐310. 36802754 10.7326/M22-1008

[32] Kaminski MF , Thomas‐Gibson S , Bugajski M , et al. Performance measures for lower gastrointestinal endoscopy: a European Society of Gastrointestinal Endoscopy (ESGE) quality improvement initiative. Endoscopy. 2017;49:378‐397. 28268235 10.1055/s-0043-103411

[33] Lund M , Erichsen R , Valori R , et al. Data quality and colonoscopy performance indicators in the prevalent round of a FIT‐based colorectal cancer screening program. Scand J Gastroenterol. 2019;54:471‐477. 30978128 10.1080/00365521.2019.1597158

[34] Larsen PT , Jørgensen SF , Hagemann‐Madsen R , Rasmussen M , Andersen B , Njor SH . Detection of colorectal cancer and advanced neoplasia during first surveillance interval after detection of adenomas in fecal immunochemical test cancer screening: a nationwide study. Endoscopy. 2024;56:853‐861. 38955210 10.1055/a-2343-5700

[35] Imperiale TF , Gruber RN , Stump TE , Emmett TW , Monahan PO . Performance characteristics of fecal immunochemical tests for colorectal cancer and advanced adenomatous polyps a systematic review and meta‐analysis. Ann Intern Med. 2019;170:319‐329. 30802902 10.7326/M18-2390

[36] Bülow Therkildsen S , Larsen PT , Njor S . Subsequent participation in organized FIT based screening following screen‐derived colonoscopy: a Danish nationwide cohort study. Prev Med Rep. 2023;32:102125. 36816764 10.1016/j.pmedr.2023.102125 PMC9929440

